# Supplementary material for: How can HIV self-testing facilitate increased access to HIV testing among multiply marginalised populations? Perspectives from GBMSM and trans women in England and Wales
Source: PLoS One. 2024 Oct 29;19(10):e0312897. doi: 10.1371/journal.pone.0312897 (PMC11521265; doi:10.1371/journal.pone.0312897)
Supplement: Attachment — Submitted filename: Response to Reviewer_150824.docx [file pone.0312897.s001.docx]

**Response to Reviewers**

15 Aug 2024

Manuscript ID: PONE-D-24-15187

Manuscript title: How can HIV self-testing facilitate increased access to HIV testing among multiply marginalised populations? Perspectives from GBMSM and trans women in England and Wales

Dear Reviewers:

Please find our itemised responses to your requests/comments. All line numbers and pages indicated below are based on the file named 'Manuscript' (i.e., the revised manuscript without track changes).

**Introduction**

Q1. The introduction should define "marginalized" in the context of their research. This would enable the reader to define the study population as early as the introduction. What qualifies as marginalized?

Answer: We appreciate your suggestion and question. We have added a context-specific definition of marginalised populations with a contextual reference on Lines 62-64, Page 5 as “marginalised populations, including people disproportionately facing discrimination or exclusion due to their ethnicity, gender/sexual identity and sexual orientation [1].” We have also provided a detailed definition of marginalised populations in the Methods section on Lines 140-142, Page 9.

Q2. The introduction should contain local and international studies on qualitative assessment of HIVST among the same or similar population. This would enhance the background of the study and contextualize the current landscape on HIVST.

Answer: Thank you for your kind advice. To better contextualise the current landscape of qualitative assessment of HIVST among GBMSM and trans populations, we have added a sentence supported by three in-text citations on Lines 105-107, Page 6: “Qualitative studies from Argentina, the Philippines and the UK also suggest that trans women may seek HIVST for privacy, convenience and alleviation of perceived stigma in healthcare facilities [25–27].”

To improve clarity of this paragraph, we rephrased the subsequent sentences on Lines 112-114, Page 7 as “Understanding such intersectional challenges is key for future HIVST programmes to benefit key populations at risk of HIV in the UK or countries with similar universal health care systems [7,30].”

Q3. What does "multiply" mean in page 7 line 113?

Answer: Thank you for raising this key question. We consider the term ‘multiply marginalised’ to describe individuals whose identity is impacted by more than one commonly marginalised attribute in specific contexts. On Lines 110-112, Page 7, we added a sentence with two in-text citations to clarify our meaning of multiply marginalised as “…especially someone whose identity is impacted by more than one commonly marginalised attribute (often described as a multiply marginalised person) [28,29].”

**Methods**

Q4. Kindly define the study design used in the qualitative investigation and the reason for choosing such a design.

Answer: Thank you for your great advice. Our study employed a pragmatic qualitative design by re-analysing personal interviews in the SELPHI qualitative dataset because it is advantageous for identifying common perspectives from marginalised individuals. We have clarified our study design and rationale on Lines 118-122, Page 8 as “This study applied a pragmatic qualitative approach by re-analysing personal interviews collected from SELPHI. Throughout SELPHI and its sub-studies, we acknowledged marginalisation as an emerging theme that required a new research inquiry. Reanalysing personal interviews was advantageous for identifying potential patterns or common perspectives across individuals with various attributes of marginalisation.” Our study design and methods for data analysis are supported by Long-Sutehall et al (2011).

Q5: How did you select the study population? It is not clear how sampling procedure was done.

Answer: We are grateful for your question. We purposively sampled 38 transcripts of multiply marginalised interviewees in the SELPHI Qualitative Dataset that comprises a total of 86 interview transcripts across three SELPHI qualitative sub-studies, already undertaken. Our purposive sampling ensured a balanced representation of individuals from diverse ethnic backgrounds, transgender people and gay, bisexual and other men who have sex with men (GBMSM).

A detailed process of our sampling strategy for secondary data analysis Is provided on Lines 150-159, Page 9: “Given that all 86 interviewees in the SELPHI Qualitative Dataset were sexual and gender minorities (i.e., GBMSM and trans women as one marginalised attribute), our sampling strategy focussed on those with additional attributes of marginalisation (i.e., non-White ethnicity or low levels of formal education). This resulted in 13 interviewees from the trans sub-study, 15 interviewees from the sub-study on GBMSM in general and 10 interviewees from the ABLA sub-study, respectively. All participants in the ABLA sub-study were non-White GBMSM and most were awarded degrees, so we identified one person with a low level of education and then randomly selected nine ABLA participants to ensure a substantial representation of individuals from diverse ethnic backgrounds. In summary, our purposive sampling for data analysis yielded 38 transcripts from multiply marginalised individuals.”

Q6. It is better to have the methods section with different subheadings in order to clearly delineate one section from another.

Answer: We appreciate your great advice and have added four subheadings in the Methods section (i.e., Study design and rationale, Data source, Sampling strategy for data analysis and Data analysis) to enhance readability.

Q7: I reckoned the authors re-analyzed the transcripts from a trial. However, it might be better elaborate more on how these transcripts were collected and re-analyzed.

Answer: Thank you for your advice. We acknowledge the importance of your request by describing how personal interview data in the SELPHI Qualitative Dataset were collected and reanalysed on Lines 131-135, Page 8: “All authors (except for IYC) were involved in the data collection of SELPHI and its sub-studies. EJN, PS, TCW and TW conducted personal interviews either online or in-person in English with audio recordings. Each recording was transcribed verbatim, anonymised and de-identified from SELPHI trial participants. The details of SELPHI’s protocol, trial outcomes and the Qualitative Dataset have been published elsewhere [18,24,32–34].”

Q8. The type of HIV self-testing was not clearly stated.

Answer: Thank you for your comment. The type of HIV self-testing used in the SELPHI trial was blood-based BioSURE® HIV Self Test Kit. We have clarified this by adding a sentence on Line 130, Page 8 as “These 86 individuals were purposively sampled from all 10,135 SELPHI participants (offered blood-based BioSURE® HIV Self Test Kit) consenting to qualitative sub-studies.”

**Results**

Q9: The results were well-written and structured. However, it might be better if a summary of the main themes using a table or figure should be included in order to clear present the results of the analysis.

Answer: We appreciate your advice. To summarise the key themes and findings of our analysis while strengthening the connection between Results and Discussion, we added **Table 2. Summary of key findings** on Line 374, Page 21. We believe this summary Table can facilitate readers’ comprehension of both Results and Discussion sections.

Q10: Page 11, line 176: you mentioned "most". How many were this "most"? Kindly qualify by including (n=X) in the statement.

Answer: Thank you for your question. We fully understand your request, but giving a precise number in this qualitative study is not advisable. During our qualitative analysis, almost every transcript mentioned some type of barriers to accessing HIV testing services. Our statement represents a general finding across the analysed transcripts. To improve clarity, we have replaced the term “Most interviewees” with “The majority of interviewees” on both Line 196, Page 12 and Line 47, Page 3 in the Abstract section.

**Discussion and Conclusions**

Q11: I want to see how the discussion and conclusion will navigate based on the concept map generated from the study results and how these themes relate to another. I am having difficulty navigating these sections.

Answer: Thank you for your suggestions. We have generated Table 2 to summarise the key themes/findings as well as help the reader navigate the Discussion section. To relevance and readability of Discussion section, we have reordered the paragraphs based on the order of themes in Table 2. We believe that the new paragraph order (Kit usability 🡺 Potential roles of HIVST in HIV testing services 🡺 Priority beneficiaries of HIVST) aligns with a clear thematic navigation illustrated in Table 2.

Q12. Are there epidemiological data of marginalized GBMSM and trans women in the country? I suggest to include these data and how the results of the study may help in easing the access of HIVST among these identified sets of population.

Answer: To our best knowledge, no epidemiological data on representative samples of marginalised GBMSM and trans women are available in the UK. Most surveillance data are categorised by single factor of marginalisation (e.g., gender identity, sexual orientation or ethnicity). We have highlighted this crucial evidence gap on Lines 422-424, Page 23-24 as “It is worth noting that, even in the UK, no epidemiological data on HIV testing uptake and/or outcomes are routinely reported for multiply marginalised populations.”

**Additional revision made by authors:**

- The content of Table 1 is revised by fully presenting the number of participants from each ethnic minority group and correcting typos.
- To improve readability, we have rephrased Lines 87-89, Page 6 as “Since 2019, HIVST has been recommended by the World Health Organization [17]. Its feasibility and acceptability in the UK has been established by the HIV Self-Testing Public Health Intervention (SELPHI) trial [18].”
- We have double-checked that all references are updated with correct formats. The number of references has increased from 44 to 52 given the requests above.
- All formatting issues in this manuscript have been tackled using PLOS ONE’s manuscript body formatting guidelines.
