# Supplementary material for: How can HIV self-testing facilitate increased access to HIV testing among multiply marginalised populations? Perspectives from GBMSM and trans women in England and Wales
Source: PLoS One. 2024 Oct 29;19(10):e0312897. doi: 10.1371/journal.pone.0312897 (PMC11521265; doi:10.1371/journal.pone.0312897)
Supplement: Attachment — Submitted filename: Response to Reviewer_091024.docx [file pone.0312897.s002.docx]

**Response to Reviewers**

09 Oct 2024

Manuscript ID: PONE-D-24-15187

Manuscript title: How can HIV self-testing facilitate increased access to HIV testing among multiply marginalised populations? Perspectives from GBMSM and trans women in England and Wales

Dear Reviewers:

Please find our responses to your comment as follows. The line number and page indicated are based on the file named 'Manuscript' (i.e., the revised manuscript without track changes).

Question 1: The term "pragmatic qualitative design" is not a standard or widely recognized qualitative research design. To ensure clarity and adherence to qualitative research methodologies, it would be beneficial for the authors to explicitly state the specific qualitative research design employed in the study (e.g., phenomenology, grounded theory, case study, etc.). This will not only provide methodological transparency but also align the study with established research frameworks in qualitative inquiry.

Answer 1: We appreciate your advice on clarifying the qualitative research design applied in this study. To explicit state our study design, we have revised the sentence on Line 118, Page 8 as 'This study applied an interpretive research design by re-analysing personal interviews collected from SELPHI.'

**Additional revision made by authors:**

- We have ensured that all in-text quotations in the Results sections are correctly formatted.
- We have revised the References section to ensure that all references are aligned with PLOS One’s reference style.
- We have updated the corresponding email of IYC.
